# Oral branched-chain amino acid granules improve structure and function of human serum albumin in cirrhotic patients

**DOI:** 10.1007/s00535-016-1281-2

**Published:** 2016-11-21

**Authors:** Hiroko Setoyama, Motohiko Tanaka, Kohei Nagumo, Hideaki Naoe, Takehisa Watanabe, Youko Yoshimaru, Masakuni Tateyama, Masato Sasaki, Hiroshi Watanabe, Masaki Otagiri, Toru Maruyama, Yutaka Sasaki

**Affiliations:** 1 Department of Gastroenterology and Hepatology, Graduate School of Medical Sciences, Kumamoto University, 1-1-1 Honjo, Chuou-ku, Kumamoto City, Kumamoto 860-8556 Japan; 2 Department of Biopharmaceutics, School of Pharmacy, Graduate School of Pharmaceutical Sciences, Kumamoto University, Kumamoto City, Kumamoto 862-0973 Japan; 3 Kumamoto Rosai Hospital, Yatsushiro City, Kumamoto 866-0826 Japan; 4 Faculty of Pharmaceutical Sciences and DDS Research Institute, Sojo University, Kumamoto City, Kumamoto 860-0082 Japan

**Keywords:** Structure–function relationship, Antioxidant, Oxidative stress, Biomarker

## Abstract

**Background and aims:**

The aim of this study was to evaluate structural and functional alterations of human serum albumin (HSA), with a special focus on the oxidized and reduced forms, in patients with chronic liver disease. We also investigated whether oral branched-chain amino acid (BCAA) supplementation could induce structural changes and improve the functions of HSA.

**Methods:**

The proportion of reduced and oxidized HSA was determined in 16 healthy controls and in 20 chronic hepatitis and 100 cirrhotic patients with stable conditions. To evaluate the functional properties of HSA, this study focused on the antioxidant and binding functions. The radical scavenging activity and binding ability of purified HSA were measured in 68 participants. After BCAA administration for 6 months, 29 patients were evaluated for HSA structural changes, with 19 out of the 29 patients also analyzed for HSA functional changes.

**Results:**

There was a significant decrease in the amounts of reduced HSA in conjunction with liver disease progression. Receiver operating characteristic curve analysis demonstrated that the levels of reduced HSA had high accuracy in determining disease progression. Functional alterations were strongly correlated to the levels of reduced HSA. BCAA supplementation led to substantial increases in the amount of reduced HSA. The altered HSA was able to scavenge significantly more radicals and restore the binding ability.

**Conclusion:**

This study describes structural alterations and functional disturbances of HSA in patients with chronic liver disease, and indicates that the levels of reduced HSA might reflect disease progression and the functional properties of HSA. Moreover, oral BCAA supplementation increases the amount of reduced HSA, thereby leading to the restoration of HSA function in cirrhotic patients.

**Electronic supplementary material:**

The online version of this article (doi:10.1007/s00535-016-1281-2) contains supplementary material, which is available to authorized users.

## Introduction

Human serum albumin (HSA) is the most abundant plasma protein, and has been considered to play a physiological role as a plasma expander [1]. Emerging evidence indicates that albumin has a variety of biological functions, including the transport of endogenous and exogenous substances, and the maintenance of colloid osmotic pressure [2]. Moreover, since extracellular fluids contain only small amounts of antioxidant enzymes, it has been proposed that circulating albumin may play a crucial role as a major antioxidant in the plasma [3, 4].

**Fig. 1:**
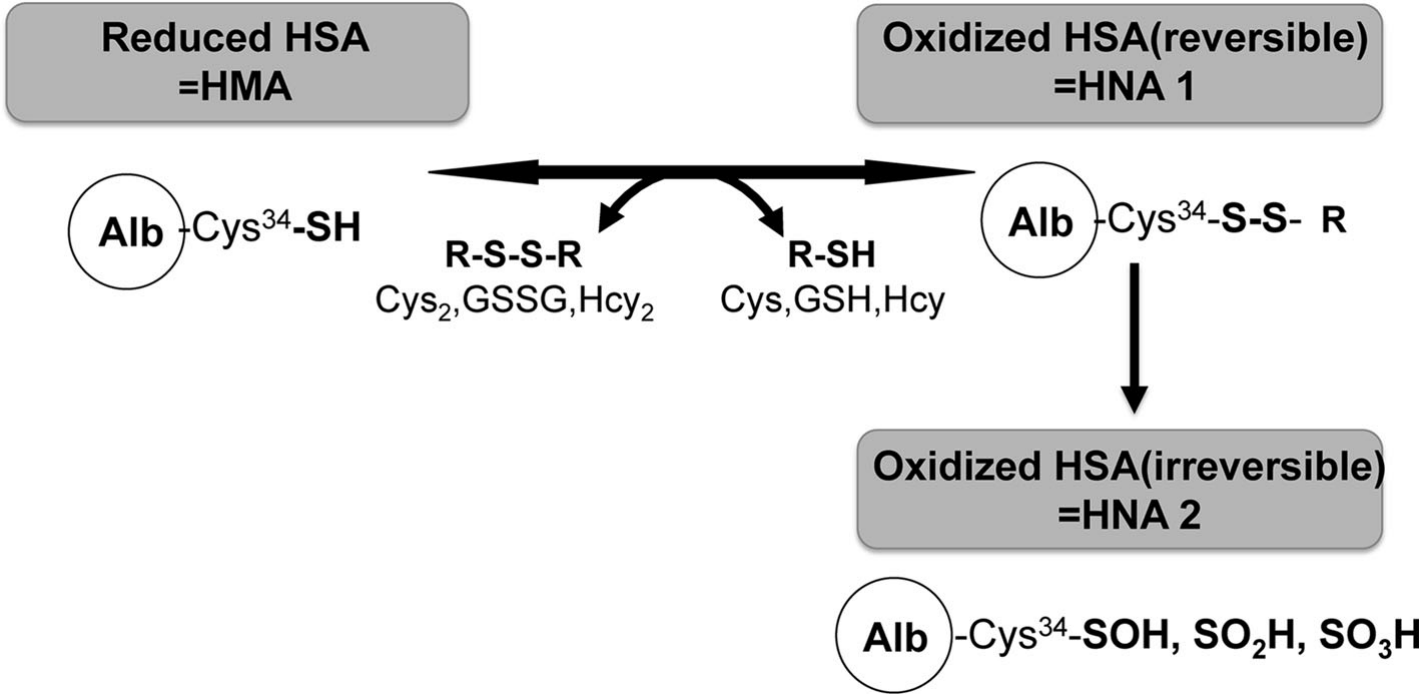
Redox states of human serum albumin (*HSA*). Depending on its redox state, plasma HSA can be divided into reduced HSA (human mercaptoalbumin, *HMA*) and oxidized HSA (human nonmercaptoalbumin, HNA). Oxidized HSA (HNA) is a mixture of the reversibly and irreversibly oxidized forms. Reversibly oxidized HSA (*HNA 1*) has mixed disulfide bonds with a thiol compound such as cysteine, homocysteine, or glutathione in the blood. In irreversibly oxidized HSA (*HNA 2*), the free thiol group is more highly oxidized to sulfenic acid (–SOH), sulfinic acid (–SO_2_H), and sulfonic acid (–SO_3_H). *Alb* albumin, *Cys* cysteine, *GSH* glutathione *GSSG* oxidized glutathione, *Hcy* homocysteine

On the other hand, because of post-translational modifications such as nitration, glycation, and oxidation, HSA exhibits structural microheterogeneity. These modifications are known to regulate the functional diversity of albumin. Furthermore, HSA is a single-chain polypeptide of 585 amino acid residues and has 17 intra-disulfide bonds and one free thiol group at cysteine, which is the 34th amino acid from the N-terminal end (Cys-34) [5, 6]. Thus, depending on the oxidative modification on Cys-34, plasma HSA is divided into reduced HSA, known as human mercaptoalbumin (HMA), and oxidized HSA, known as human nonmercaptoalbumin (HNA) (Fig. 1).

In healthy adults, approximately 75% of the Cys-34 molecules in HSA contain a free sulfhydryl group (reduced HSA, HMA), and approximately 25% of Cys-34 forms a reactive disulfide bond with a sulfurous amino acid (oxidized HSA, HNA) [7]. HNA is a mixture of the reversible (HNA1) and irreversible (HNA2) oxidized forms. HNA1 has mixed disulfide bonds with a thiol compound such as cysteine, homocysteine, or glutathione. In contrast, the free thiol group is more highly oxidized to sulfenic acid (–SOH), sulfinic acid (–SO_2_H), and sulfonic acid (–SO_3_H) in HNA2 [8]. This has been demonstrated by high-performance liquid chromatography (HPLC) analysis [9]. Furthermore, changes in this redox state of the HSA have been reported in the elderly, with physical exercise, in various pathophysiological conditions such as diabetes mellitus and renal and hepatic dysfunctions, and in patients under anesthesia during surgery [10–12].

Oettl et al. [13] reported that the fraction of oxidized HSA, especially HNA2, increased in patients with acute-on-chronic liver failure (ACLF). In stable cirrhotic patients without acute exacerbation, the synthesis and degradation rates of HSA are both especially decreased, and the biological half-life of HSA is prolonged [14]. In addition, in chronic liver disease, oxidative stress is abundant in both the intracellular fluid and extracellular fluid. Therefore, the ratio of oxidized HSA to total HSA increases with the progression of liver disease [15].

Watanabe et al. [7] examined cirrhotic patients and analyzed the microheterogeneity of HSA, including oxidized HSA, reduced HSA, and glycoalbumin, and found that although there was an elevated ratio of oxidized HSA to total HSA, there was also a decrease in the amount of total HSA. However, the precise mechanism responsible for the increased ratio of oxidized HSA to total HSA remains to be elucidated.

In addition to the studies on the quantity of HSA, the quality of HSA has also been examined. Kawakami et al. [16] have clarified the structure–function relationship between reduced and oxidized HSA in healthy human plasma. They also reported that oxidation in terms of S-cysteinylation at Cys-34 in vitro could result in a number of regional structural changes of HSA associated with alterations of the functional properties, including antioxidation and ligand-binding affinities.

Within this context, the previous studies have described disturbances in the antioxidant function and binding activity, and in the transportation capacity of HSA in patients with ACLF [17] and with acute deterioration of cirrhosis [18]. However, little is known about the functional alterations of HSA that occur with gradual progression of chronic liver disease without acute exacerbation. Since functional changes of HSA are expected to be closely related to structural changes of HSA, we evaluated the structural change along with two functional properties (i.e., the antioxidant function and the binding capacity) of HSA in patients with chronic liver diseases.

On the other hand, oral supplementation with branched-chain amino acids (BCAA) is widely used and has been demonstrated to have clinical efficacy in relieving hypoalbuminemia in patients with decompensated cirrhosis [19, 20]. Orally administrated BCAA augments albumin synthesis in the liver, not only by supplementation of material substrates for protein synthesis, but also by induction of a mammalian target of rapamycin signal that is critical for translation initiation [21, 22]. A recent animal study has reported that continuous supplementation of BCAA in rats with chronic liver disease induced phosphorylation of the hepatic ribosomal protein S6 and prevented a decrease in amount of the reduced HSA [23]. Furthermore, it has been reported that oral BCAA supplementation can increase the decreased synthesis and degradation rates and reverse the changes in the ratio of oxidized HSA to reduced HSA [14, 15]. Therefore, our study also investigated whether BCAA supplementation can induce structural changes that lead to improvement of the function of HSA.

## Methods

### Participants

Patients who were treated for chronic liver disease at Kumamoto University Hospital or Kumamoto Rosai Hospital from September 2009 to March 2013 were enrolled in this study. HSA structural changes were examined in 136 participants, including 16 healthy controls, 20 chronic hepatitis (CH) patients, and 100 cirrhotic patients. Chronic liver disease was diagnosed on the basis of clinical and laboratory profiles. The causes of chronic liver disease were as follows: hepatitis B virus (HBV) infection in 7 patients, hepatitis C virus (HCV) infection in 74 patients, alcoholic liver injury in 17 patients, and other causes (nonalcoholic steatohepatitis, primary biliary cirrhosis, etc.) in 22 patients. To measure the serum albumin level, we used the modified bromocresol purple method [24]. Hematologic and biochemical tests were performed with standard laboratory techniques. Since the present study focused on chronic liver disease patients who were currently stable, we excluded patients with ACLF. The healthy controls matched the chronic liver disease patients with regard to age. The cirrhotic patients were stratified according to the Child–Pugh classification [25], with 30 patients classified as having Child–Pugh class A (CP-A) cirrhosis, 40 patients classified as having Child–Pugh class B (CP-B) cirrhosis, and 30 patients classified as having Child–Pugh class C (CP-C) cirrhosis. Table 1 summarizes the profiles of the healthy controls and the chronic liver disease patients. At the time of enrollment, none of the patients had received any prior oral BCAA supplementation therapy. Of the 136 participants, 68 were randomly selected and analyzed for HSA functional changes. This group included 9 healthy controls, 10 chronic hepatitis patients, and 49 cirrhotic patients (15 patients with CP-A cirrhosis, 20 patients with CP-B cirrhosis, and14 patients with CP-C cirrhosis).

**Table 1:** Demographic characteristics of the subjects

	Control	CH	LC	LC		
	(*n* = 16)	(*n* = 20)	(*n* = 100)	Child A (*n* = 30)	Child B (*n* = 40)	Child C (*n* = 30)
Age	61.8 ± 3.5	58.2 ± 10.9*	64.8 ± 10.8^‡^	66.6 ± 10.6	66.2 ± 9.5	61.1 ± 12.0
Sex (Male/Female)	7/9	10/10	58/42	17/13	24/16	17/13
Alb (g/dL)	4.38 ± 0.27*	3.99 ± 0.37*	3.10 ± 0.56^†,‡^	3.65 ± 0.46^b,c^	3.00 ± 0.35^a,c^	2.70 ± 0.42^a,b^
AST (IU/L)	22.3 ± 4.5*	41.4 ± 23.7	65.9 ± 49.5^†^	60.3 ± 33.9	66.1 ± 47.3	71.1 ± 64.3
ALT (IU/L)	17.7 ± 4.9*	45.1 ± 32.0	47.5 ± 37.0^†^	49.9 ± 30.5	52.8 ± 39.8	38.0 ± 38.5
T.Bil (mg/dL)	0.53 ± 0.25*	0.80 ± 0.30	2.08 ± 2.53^†^	0.97 ± 0.44^c^	1.44 ± 0.75^c^	4.04 ± 3.89^a,b^
Prothrombin activity (%)	–	96.8 ± 11.5*	71.3 ± 18.0^‡^	88.6 ± 12.2^b,c^	70.5 ± 14.4^a,c^	55.9 ± 11.4^a,b^
NH3 (μg/dL)	–	46.8 ± 15.2*	72.2 ± 41.3^‡^	51.6 ± 21.4^b,c^	82.2 ± 42.0^a^	81.9 ± 48.5^a^
BTR	7.00 ± 0.51*	6.20 ± 1.47*	3.58 ± 1.97^†,‡^	4.35 ± 1.54	2.94 ± 0.97	3.15 ± 2.92

Values are expressed as mean ± SD (standard deviation).* P* < 0.05 by ANOVA

*CH* Chronic hepatitis,* LC* Liver cirrhosis,* Alb* Albumin,* AST* Aspartate aminotransferase,* ALT* Alanine aminotransferase,* T.Bil* Total bilirubin, * BTR* Branched chain amino acid to tyrosine ratio

* Significantly different versus LC (*P* < 0.05)

† Significantly different versus Control (*P* < 0.05)

‡ Significantly different versus CH (*P* < 0.05)

^a^Significantly different versus Child A (*P* < 0.05)

^b^Significantly different versus Child B (*P* < 0.05)

^c^Significantly different versus Child C (*P* < 0.05)

To determine the effects of BCAA supplementation, 29 cirrhotic patients (10 patients with CP-A cirrhosis, 13 patients with CP-B cirrhosis, and 6 patients with CP-C cirrhosis) were given 4 g BCAA granules orally (Livact^®^ Granules, Ajinomoto Pharmaceuticals, Tokyo, Japan; one sachet contained 4.15 g BCAA: 952 mg l-isoleucine, 1904 mg l-leucine, and 1144 mg l-valine) after each meal for 6 months (Table S1 summarizes the profiles of the patients treated with BCAA). All patients were evaluated for HSA structural changes, with 19 out of the 29 patients (5 patients with CP-A cirrhosis, 10 patients with CP-B cirrhosis, and 4 patients with CP-C cirrhosis) also analyzed for HSA functional changes.

Written informed consent was obtained from each participant. The study protocol conformed to the guidelines of the 2008 Declaration of Helsinki, and was approved by the Ethics Review Board for Human Research at Kumamoto University (approval no. 581, issued on August 8, 2009).

### Analysis of redox state of HSA

HPLC was performed as described by Hayashi et al. [5]. On the basis of the HPLC profiles of HSA, the values of each of the albumin fractions (those of HMA, HNA1, and HNA2) were estimated by division of the area of each fraction by the total area corresponding to HSA. We defined HMA as reduced HSA, and we defined HNA (i.e., mixture of HNA1 and HNA2) as oxidized HSA.

### Biological properties of HSA

#### Radical scavenging activity of HSA purified from patients

The radical scavenging activity of HSA was determined by use of 1,1-diphenyl-2-picrylhydrazyl (DPPH) radical in accordance with a previously reported method [6].

#### Evaluation of the antioxidant potential in plasma

The biological antioxidative potential in patients’ plasma was measured with a free-radical analytical system (FRAS 4; Diacron International) [26].

#### Assessment of the levels of oxidative stress metabolites

A commercially available method (derivative of reactive oxygen metabolites test; Diacron International, Grosseto, Italy) [26, 27] was used to assess the levels of oxidative stress metabolites in the blood samples.

#### Binding of ketoprofen to HSA purified from patients

The ability of purified HSA to bind ketoprofen was examined by the ultrafiltration method previously described [6].

### Statistical analysis

Data were expressed as the mean ± standard deviation. Values were considered statistically significant when *P* < 0.05. Statistical significance was evaluated by analysis of variance followed by Bonferroni’s method. We additionally confirmed the statistical significance of the detected trends by means of the nonparametric Jonckheere–Terpstra test to evaluate trends in data. Receiver operating characteristic (ROC) curves and Pearson’s correlation were also used for the statistical analyses.

Data were analyzed with IBM SPSS Statistics, version 19.0 (IBM, Armonk, NY, USA).

## Results

### Structural changes of HSA with the progression of liver disease

**Fig. 2:**
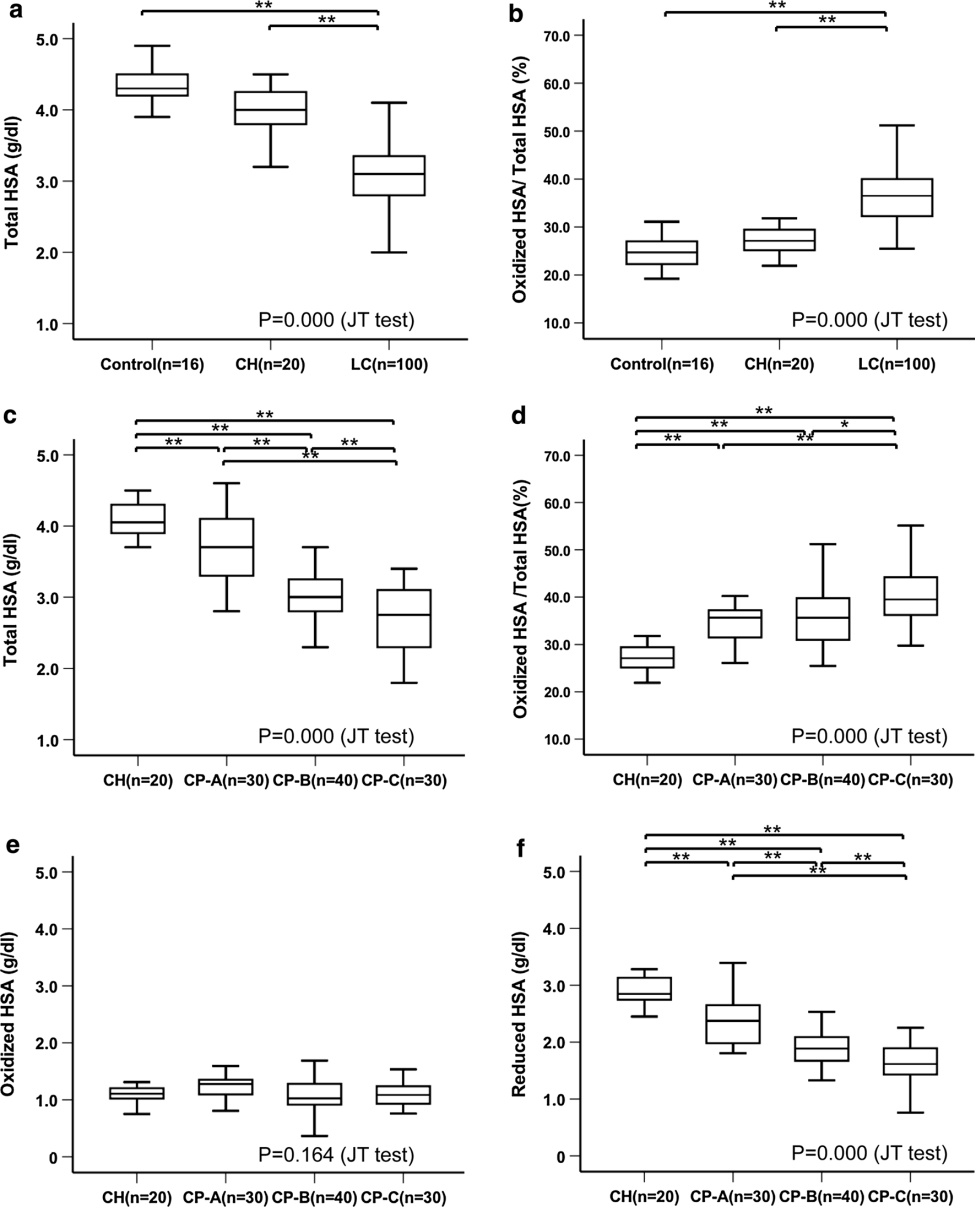
Structural changes of human serum albumin (*HSA*) in relation to the progression of liver disease. Box and whisker plots of the levels of HSA. Change in the amount of total HSA (**a**) and oxidized HSA to total HSA ratio (**b**) with the progression of liver disease. Change in the amount of total HSA (**c**), oxidized HSA to total HSA ratio (**d**), amount of oxidized HSA (**e**), and amount of reduced HSA (**f**) with the progression of liver disease with special reference to the Child–Pugh classification. Data were analyzed by a Jonckheere–Terpstra (*JT*) test and analysis of variance (ANOVA). *P* < 0.05 was considered to indicate a statistically significant difference. *P < 0.05, **P < 0.01, ANOVA. *CH* chronic hepatitis, *CP-A* Child–Pugh class A cirrhosis, *CP-B* Child–Pugh class B cirrhosis, *CP-C* Child–Pugh class C cirrhosis, *LC* cirrhosis

There was a significant decrease in the amount of total HSA with the progression of liver disease (controls, 4.38 ± 0.27 g/dl; chronic hepatitis patients, 3.99 ± 0.37 g/dl; cirrhotic patients, 3.10 ± 0.55 g/dl) (Fig. 2a). In contrast, there was a significant increase in the ratio of oxidized HSA to total HSA (controls, 24.8 ± 3.5%; chronic hepatitis patients, 27.9 ± 3.9%; cirrhotic patients, 37.3 ± 7.9%) (Fig. 2b).

When the Child–Pugh classification was applied to cirrhotic patients, a similar tendency was also observed (Fig. 2c), with a significant difference in the amount of total HSA observed for CP-A cirrhosis patients (3.65 ± 0.46 g/dl), CP-B cirrhosis patients (3.00 ± 0.35 g/dl), and CP-C cirrhosis patients (2.70 ± 0.42 g/dl). A significant increase was also seen for the ratio of oxidized HSA to total HSA when it was analyzed with regard to the Child-Pugh classification (CP-A cirrhosis patients, 34.3 ± 5.1%; CP-B cirrhosis patients, 36.6 ± 8.5%; CP-C cirrhosis patients, 41.3 ± 8.0%) (Fig. 2d). We additionally determined the amounts of oxidized and reduced HSA. Although there was no difference in the amount of oxidized HSA (chronic hepatitis patients, 1.11 ± 0.18 g/dl; CP-A cirrhosis patients, 1.24 ± 0.19 g/dl; CP-B cirrhosis patients, 1.11 ± 0.33 g/dl; CP-C cirrhosis patients, 1.10 ± 0.22 g/dl) (Fig. 2e), there was a significant decrease in the amount of reduced HSA with the progression of liver disease (chronic hepatitis patients, 2.87 ± 0.32 g/dl; CP-A cirrhosis patients, 2.40 ± 0.43 g/dl; CP-B cirrhosis patients, 1.90 ± 0.29 g/dl; CP-C cirrhosis patients, 1.60 ± 0.36 g/dl) (Fig. 2f).

Although there was only a weak correlation between the amount of total HSA and the amount of oxidized HSA in patients with chronic liver disease (Pearson correlation coefficient 0.291) (Fig. S1A), there was a very strong correlation between the amount of total HSA and the amount of reduced HSA (Pearson correlation coefficient 0.930) (Fig. S1B). These findings indicate that the reduction in the total HSA amount could be accounted for by the decrease in the amount of reduced HSA.

ROC analysis showed that the levels of reduced HSA associated with disease progression exhibited the highest areas under the ROC curves (AUROC curves) for all parameters (concentration of total HSA, concentration of reduced HSA, concentration of oxidized HSA, and the ratio of the concentration of oxidized HSA to the concentration of total HSA) (Table 2). For the levels of reduced HSA, the optimum cutoff values used to distinguish chronic hepatitis patients from healthy controls was 3.11 g/dl (AUROC curve 0.903; *P* < 0.001, sensitivity 75%, specificity 100%), and 2.67 g/dl (AUROC curve 0.931; *P* < 0.001, sensitivity 93%, specificity 85%) for separating cirrhosis from chronic hepatitis. The optimum cutoff values for the levels of reduced HSA to distinguish CP-A cirrhosis from chronic hepatitis, CP-B cirrhosis from CP-A cirrhosis, and CP-B cirrhosis from CP-C cirrhosis were 2.65 g/dl (AUROC curve 0.813; *P* < 0.001, sensitivity 77%, specificity 90%), 2.11 g/dl (AUROC curve 0.837; *P* < 0.001, sensitivity 78%, specificity 70%), and 1.71 g/dl (AUROC curve 0.740; *P* < 0.001, sensitivity 63%, specificity 73%) respectively (Table S2). Since the AUROC curve of the asparate aminotranferase to platelet ratio index is known to be a hepatic fibrosis marker [28], this parameter could be used to largely discriminate cirrhosis from chronic hepatitis (AUROC curve 0.760; *P* < 0.001, sensitivity 79%, specificity 63%). However, it should be noted that the discriminating abilities of the levels of reduced HSA were higher than those of the asparate aminotranferase to platelet ratio index, especially for distinguishing the different Child–Pugh cirrhosis classes (AUROC curve ranging from 0.51 to 0.52).

**Table 2:** Area under the receiver operating characteristic (*AUROC*) curve for each parameter with chronic liver disease progression

	Controls → CH patients			CH patients → Cirrhotic patients		
	AUROC curve	95% CI	*P*	AUROC curve	95% CI	*P*
Total HSA (g/dl)	0.800	0.657–0.943	0.002	0.898	0.832–0.963	0.000
Oxidized/total HSA	0.722	0.551–0.892	0.024	0.894	0.820–0.969	0.000
Oxidized HSA (g/dl)	0.598	0.406–0.791	0.316	0.51	0.423–0.659	0.567
Reduced HSA (g/dl)	0.903	0.807–0.999	0.000	0.931	0.875–0.987	0.000

*P* < 0.05 was considered to indicate a statistically significant difference

*CH* chronic hepatitis, *CI* confidential interval, *HSA* human serum albumin

### Functional changes of HSA with the progression of liver disease

**Fig. 3:**
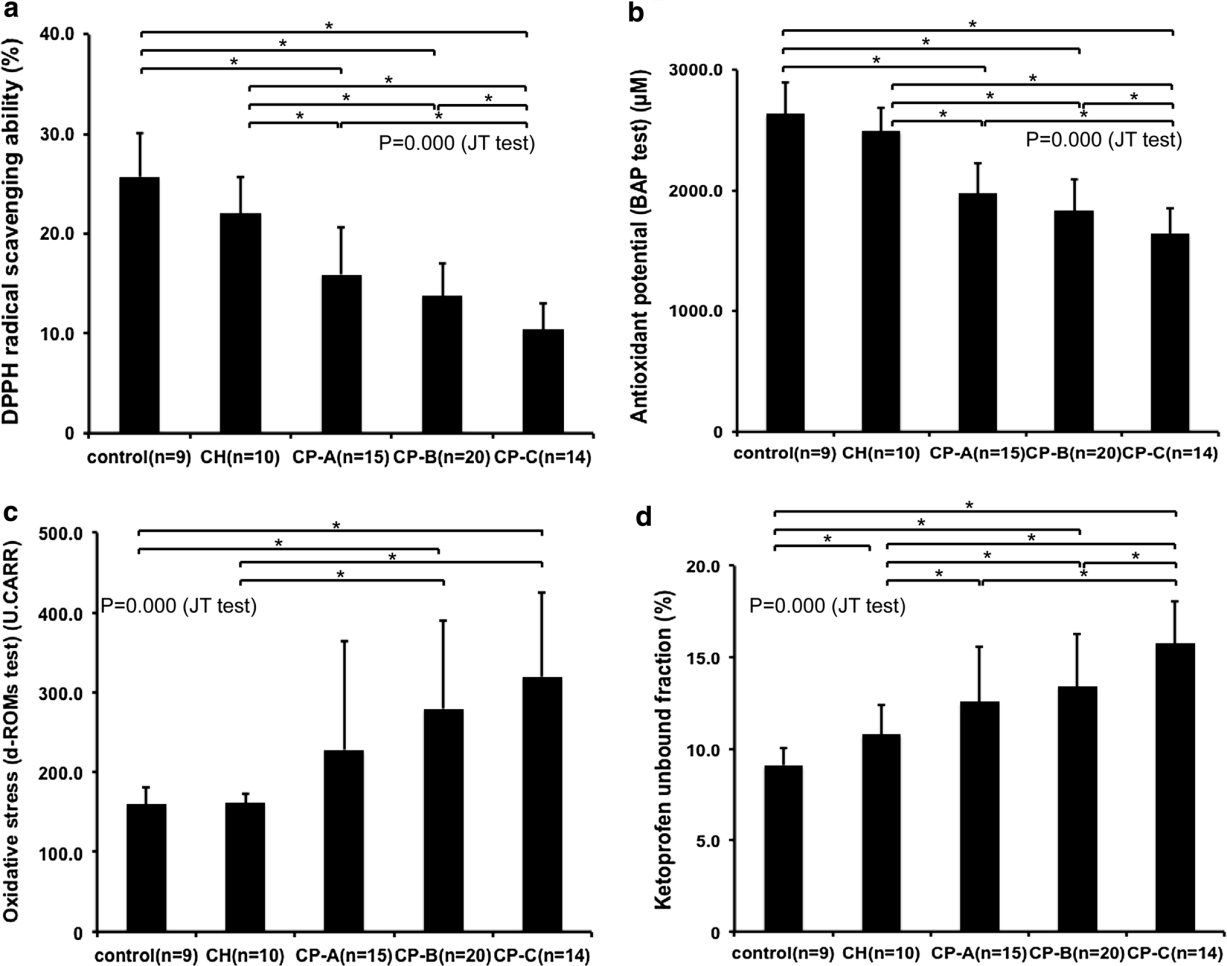
Functional changes of human serum albumin (HSA) with the progression of liver disease **a** 1,1-Diphenyl-2-picrylhydrazyl (*DPPH*) radical scavenging ability of purified HSA. **b** Antioxidant potential of plasma—biological antioxidant potential (*BAP*) test. **c** Levels of oxidative stress metabolites in the plasma—derivative of reactive oxygen metabolites (*d-ROMs*) test. **d** Unbound fraction of ketoprofen to purified HSA. Values are expressed as the mean ± standard deviation. Data were analyzed by a Jonckheere–Terpstra (*JT*) test and analysis of variance (ANOVA). *P* < 0.05 was considered to indicate a statistically significant difference. *P < 0.05, ANOVA. *CH* chronic hepatitis, *CP-A* Child–Pugh class A cirrhosis, *CP-B* Child–Pugh class B cirrhosis, *CP-C* Child–Pugh class C cirrhosis, *U. CARR* Carratelli units

The DPPH radical scavenging ability of HSA purified from the patients was significantly disturbed in the CP-A cirrhosis, CP-B cirrhosis, and CP-C cirrhosis patients as compared with the healthy controls and chronic hepatitis patients (controls, 25.7 ± 4.4%; chronic hepatitis patients, 22.1 ± 3.7%; CP-A cirrhosis patients, 15.9 ± 4.7%; CP-B cirrhosis patients, 13.8 ± 3.2%; CP-C cirrhosis patients, 10.4 ± 2.6%). The radical scavenging ability in the CP-C cirrhosis patients as compared with the CP-A cirrhosis and CP-B cirrhosis patients was also significantly disturbed (Fig. 3a).

The antioxidant potential of the cirrhotic patients were also shown to be significantly disturbed (controls, 2639.5 ± 255.9 μM; chronic hepatitis patients, 2494.6 ± 197.6 μM; CP-A cirrhosis patients, 1976.2 ± 251.6 μM; CP-B cirrhosis patients, 1833.1 ± 261.7 μM; CP-C cirrhosis patients, 1645.6 ± 210.3 μM) (Fig. 3b). The antioxidant potential in CP-C cirrhosis patients was also significantly lower than that in CP-A cirrhosis and CP-B cirrhosis patients (Fig. 3b). The levels of the oxidative stress metabolites (reactive oxygen metabolites) in the cirrhotic patients as compared with the healthy controls and chronic hepatitis patients were significantly elevated [controls, 159.6 ± 21.6 Carratelli units (U.CARR); chronic hepatitis patients, 161.5 ± 11.6 U.CARR; CP-A cirrhosis patients, 228.3 ± 136.0 U.CARR; CP-B cirrhosis patients, 279.7 ± 109.7 U.CARR; CP-C cirrhosis patients, 319.7 ± 104.7 U.CARR] (Fig. 3c).

There was also a significant increase in the unbound fraction of ketoprofen in the chronic liver disease patients compared with the healthy controls. This increase occurred in association with liver disease progression (controls, 9.1 ± 1.0%; chronic hepatitis patients, 10.8 ± 1.6%; CP-A cirrhosis patients, 12.6 ± 3.0%; CP-B cirrhosis patients, 13.4 ± 2.8%; CP-C cirrhosis patients, 15.8 ± 2.3%) (Fig. 3d).

Each of the functional properties exhibited a moderate to strongly significant correlation with the amounts of reduced HSA (Fig. S2): strong positive correlations were especially observed between the amount of reduced HSA and the DPPH radical scavenging activity (Pearson correlation coefficient 0.734) (Fig. S2A) or antioxidant potential (Pearson correlation coefficient 0.736) (Fig. S2B).

### The effects of BCAA supplementation on HSA

**Fig. 4:**
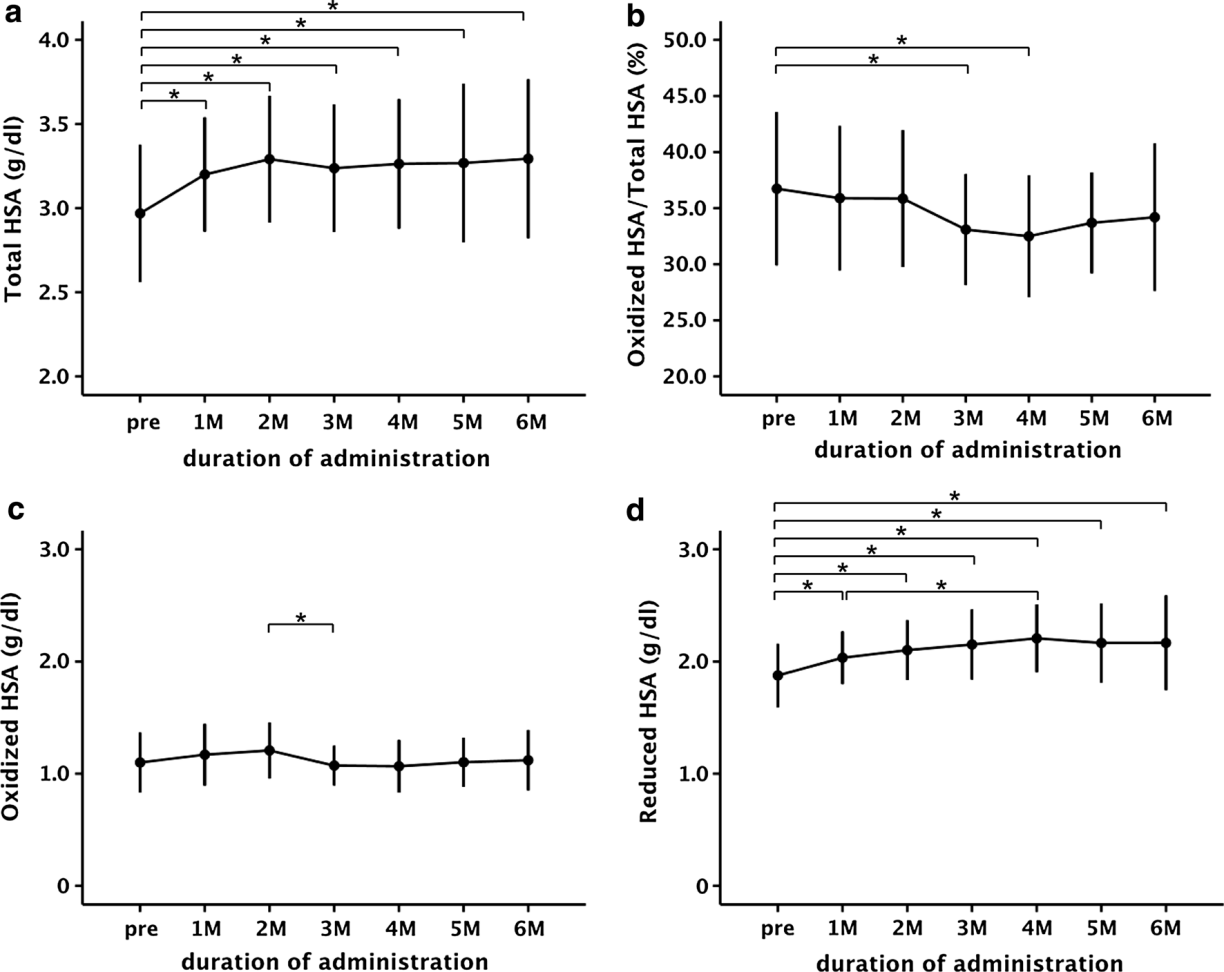
Structural changes of human serum albumin (*HSA*) induced by oral branched-chain amino acid (BCAA) supplementation in cirrhotic patients (*n* = 29). Alteration of the amount of total HSA (**a**), oxidized albumin to total albumin ratio (**b**), amount of oxidized HSA (**c**), and amount of reduced HSA (**d**) induced by oral BCAA supplementation. Values are expressed as the mean ± standard deviation. *P* < 0.05 was considered to indicate a statistically significant difference. *P < 0.05, analysis of variance. *M* month(s), *pre* pretreatment

BCAA supplementation significantly increased the amount of total HSA (Fig. 4a). Although there was a significant decrease in the ratio of oxidized HSA to total HSA, there was little difference in the amount of oxidized HSA (Fig. 4b, c). By contrast, there was a significant increase in the amount of reduced HSA after BCAA supplementation (Fig. 4d).

**Fig. 5:**
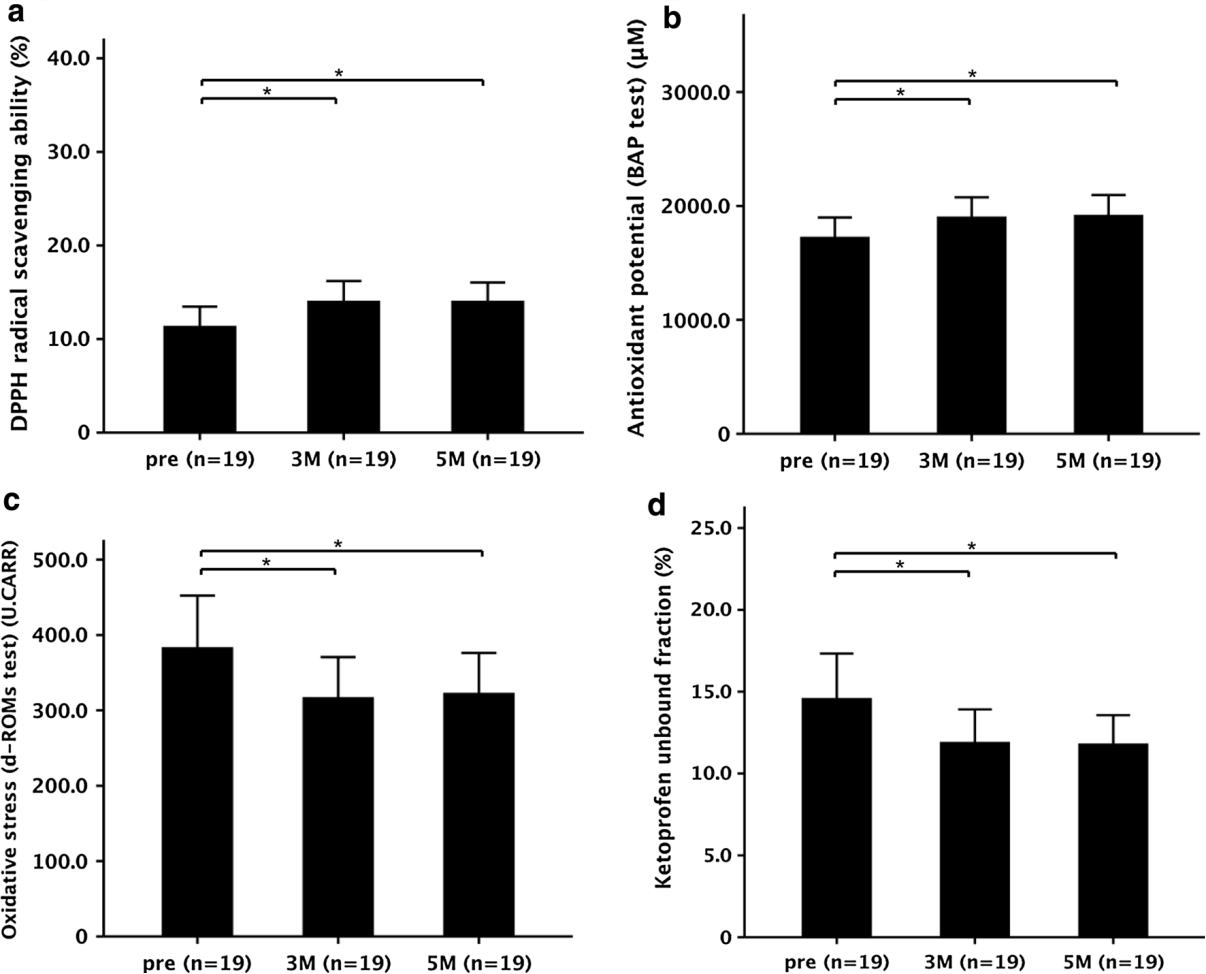
Functional changes of human serum albumin (HSA) induced by oral branched-chain amino acid (BCAA) supplementation. **a** 1,1-Diphenyl-2-picrylhydrazyl (*DPPH*) radical scavenging ability of purified HSA. **b** Antioxidant potential of plasma—biological antioxidant potential (*BAP*) test. **c** Levels of oxidative stress metabolites in plasma—derivative of reactive oxygen metabolites (*d-ROMs*) test. **d** Unbound fraction of ketoprofen to purified HSA. Values are expressed as the mean ± standard deviation. Data were analyzed by analysis of variance (ANOVA). *P* < 0.05 was considered to indicate a statistically significant difference. *P < 0.05, ANOVA. *3* *M* 3 months of treatment, *5* *M* 5 months of treatment, *pre* pretreatment, *U.CARR* Carratelli units

DPPH radical scavenging ability after 3 months of treatment (14.0 ± 2.1%) and after 5 months of treatment (14.0 ± 2.0%) was significantly increased as compared with the pretreatment levels (11.3 ± 2.1%) (Fig. 5a). In addition, there was a significant increase of the antioxidant potential between the pretreatment level (1724.5 ± 173.9 μM) and levels at 3 months (1903.3 ± 172.9 μM) and 5 months (1918.1 ± 177.0 μM) (Fig. 5b). A significant decrease in the levels of oxidative stress metabolites was observed after 3 months (316.9 ± 53.9 U.CARR) and 5 months (322.7 ± 53.4 U.CARR) of treatment as compared with the pretreatment levels (383.2 ± 69.1 U.CARR) (Fig. 5c).

The ketoprofen unbound fraction decreased significantly after 3 months (11.9 ± 2.0%) and 5 months (11.8 ± 1.8%) of treatment as compared with the pretreatment levels (14.6 ± 2.7%) (Fig. 5d). However, the antioxidant and binding functions in the cirrhotic patients were never able to recover to control levels, even after 5 months of BCAA supplementation (Figs. 3a, b, d, 5a, b, d).

No significant changes were noted in the clinical parameters such as the prothrombin time (Fig. S3A), serum total bilirubin concentration (Fig. S3B), and Child–Pugh scores (Fig. S3C) after the BCAA supplementation. Change in homeostasis model assessment insulin resistance (HOMA-IR) was also analyzed in 10 of 29 patients (2 patients with CP-A cirrhosis, 6 patients with CP-B cirrhosis, and 2 patients with CP-C cirrhosis). The HOMA-IR tended to decrease after 3 months (7.0 ± 4.6) and 5 months (10.5 ± 9.2) of treatment as compared with the pretreatment levels (12.4 ± 14.1), but there was no significant difference.

## Discussion

HSA exhibits microheterogeneity in structure and function, due to post-translational nonenzymatic modifications, including nitration, glycation, and oxidation. In this context, we focused on oxidative modification of HSA in this study. The amount of oxidized HSA tends to be higher in patients with various diseases or conditions [7, 10, 17]. The current study demonstrated there was a decrease in the amount of total HSA and a significant increase in the ratio of oxidized HSA to total HSA with the progression of liver disease (Fig. 2a, b), which is supported by previous reports [7, 29]. We further showed that there was a strong correlation between the amounts of total HSA and reduced HSA (Fig. S1B). Using the levels of reduced HSA obtained by our ROC analysis, we were also able to distinguish the stages of the chronic liver disease with high sensitivity and reasonable specificity (Tables 2, S2). On the other hand, we have preliminarily compared the incidence of hepatocellular carcinoma between the group with higher levels of reduced HSA (reduced HSA concentration greater than 2.7 g/dl) (*n* = 43) and the group with lower levels (reduced HSA concentration 2.7 g/dl or lower) (*n* = 44). The cumulative incidence of hepatocellular carcinoma was significantly lower in the group with higher level that in the group with lower levels (Fig. S4). These findings lead us to speculate that reduced HSA might serve as a novel biomarker reflecting disease progression of chronic liver disease as well as hepatocarcinogenesis.

Although the significance of oxidized HSA, especially HNA2, has been described in patients with ACLF [13], we attempted to clarify the role of reduced HSA in patients with chronic stable liver disease without acute exacerbation, because the reduced form is presumed to be a dominant configuration for the functional properties of HSA.

It has previously been shown that HSA plays an important role as an antioxidant in plasma [3, 6]. In this regard, we described that the radical scavenging ability of HSA and the antioxidant potential of plasma significantly deteriorated with the severity of the chronic liver disease (Fig. 3a, b). Moreover, the serum level of the oxidative stress metabolites increased, especially in the CP-C cirrhosis patients (Fig. 3c). These observations clearly indicate a prominent disturbance of the antioxidant function of HSA in patients with advanced cirrhosis. Furthermore, these findings also suggest that increase of the antioxidant potential of HSA might lead to a reduction of oxidative stress throughout the entire body.

HSA also has a ligand binding property contributing to tissue distribution and elimination of various endogenous and exogenous substances, i.e., fatty acids, pharmaceuticals, and endotoxins [1], which accounts for its physiological roles in drug delivery, efficacy, detoxification, and antioxidant protection [2]. Oettl and Stauber [30] have reported that the redox state of HSA can modulate the binding properties via a variety of mechanisms, including alterations in the conformation that can lead to changed affinities at the binding sites, with the altered binding especially observed when the binding reaction itself is redox sensitive. In addition, previous studies have demonstrated that the ligand binding property of site II was impaired under oxidative stress conditions and the reduced binding capacity of the albumin site II is mainly related to the impaired liver function in advanced liver disease [13, 31]. These observations led us to investigate the ligand binding property of ketoprofen, which is a typical site II bound ligand. By using HSA purified from patient plasma, we found that there was a significant impairment of the binding affinity of HSA for ketoprofen in cirrhotic patients (Fig. 3d).

Since reduced HSA exhibited a moderate to strong correlation with the functional properties of HSA (Fig. S2), it can be speculated that reduced HSA might serve as a biomarker for surrogating functional HSA.

On the basis of these observations, we attempted to focus on alteration of the structure and function of HSA induced by BCAA supplementation. Regarding structural change of HSA induced by oral BCAA supplementation, the current findings indicate that BCAA supplementation could change the redox state of HSA, leading to an increase in the amount of reduced HSA (Fig. 4d). On the basis of the present limited data, it is not sufficient to explain a precise mechanism by which the BCAA supplementation enhances the level of reduced HSA. So far, there is little information regarding the redox state of HSA newly secreted from hepatocytes. In addition, there is no evidence supporting the existence of post-translational modification of Cys-34 in HSA during the transcription and secretory processes. On the other hand, Kawakami et al. [16] found that the oxidized form of Cys-34 in HSA from plasma of healthy individuals was largely cysteinylated. Additionally, Suzuki et al. [32] reported that there is a close relationship between the redox state of HSA and serum cysteine levels in nondiabetic chronic kidney disease. These findings suggest that the redox state of HSA in plasma may be regulated by the plasma cysteine levels. If this is correct, a newly secreted HSA molecule resulting from BCAA supplementation is likely to be in a reduced form. To prove the hypothesis, it is necessary to identify the redox form of HSA that is newly synthesized in hepatocytes as a result of BCAA supplementation, in terms of inducing a mammalian target of rapamycin signal. This would be an important future subject.

Because we have clarified that the antioxidant and binding functions were strongly associated with the level of reduced HSA (Fig. S2), we further investigated the influence of the BCAA supplementation on the functional alteration of HSA. The radical scavenging, antioxidant potential, and binding ability of HSA were significantly restored after BCAA supplementation in cirrhotic patients (Fig. 5). After BCAA supplementation as compared with the pretreatment period, HSA in cirrhotic patients scavenged more DPPH radicals, which was associated with the increase in the antioxidant potential and the decrease in the levels of oxidative stress metabolites in the plasma (Fig. 5a–c). With regard to the binding function of HSA, we also described a significantly restored ability to bind to ketoprofen (Fig. 5d).

Marchesini et al. [33] and Muto et al. [19] have reported that BCAA supplementation over a long period increases event-free survival, increases HSA concentration, and improves quality of life in patients with decompensated cirrhosis. Hayaishi et al. [34] also described that oral BCAA supplementation was associated with a reduced incidence of hepatocellular carcinoma in patients with cirrhosis.

On the other hand, oxidative stress has been proven to participate in the progression of liver disease and hepatocarcinogenesis [35, 36]. It has also been shown that oxidative stress plays a crucial role in the morbidity in nonalcoholic fatty liver disease [37], alcoholic liver injury [38], and hepatitis C [39]. Because HCV infection is often accompanied by iron accumulation or fatty deposition in hepatocytes, oxidative stress must participate in the morbidity in HCV-related chronic liver disease [39]. In this context, we have preliminarily determined the change in oxidative stress after HCV eradication in chronic hepatitis patients (*n* = 12). The levels of reduced HSA significantly recovered after the treatment, compared with pretreatment levels (Fig. S5), indicating that HCV eradication would prevent progression of liver disease in terms of attenuating oxidative stress.

From the findings taken together, we can speculate that BCAA supplementation could restore the antioxidative function of HSA via an increase in the level of reduced HSA, thereby leading to an improved prognosis for cirrhotic patients. In addition, since we did not observe any changes in the clinical parameters or the Child–Pugh score (Fig. S3), the level of reduced HSA might also serve as a more sensitive parameter of the BCAA supplementation efficacy.

In conclusion, the present study demonstrated that oxidative modification of HSA in patients with chronic stable liver disease could contribute to the structural and the functional alterations of HSA, and suggest that reduced HSA might be a novel discriminative biomarker of the disease progression in chronic liver disease patients. The present findings also indicate that reduced HSA could be used as a biomarker surrogating “functional albumin” in chronic liver disease patients with stable conditions.

Furthermore, oral BCAA supplementation changes the redox state of HSA, which leads to the restoration of HSA functions in decompensated cirrhosis patients. Therefore, BCAA supplementation may be beneficial for preventing the progression of serious complications and improving the prognosis of patients with chronic liver disease by maintaining the physiological functions of HSA.

## Electronic supplementary material

Below is the link to the electronic supplementary material.

Supplementary material 1 (TIF 86 kb)

Supplementary material 2 (TIF 137 kb)

Supplementary material 3 (TIF 85 kb)

Supplementary material 4 (TIF 58 kb)

Supplementary material 5 (TIF 77 kb)

Supplementary material 6 (TIF 7077 kb)

Supplementary material 7 (TIF 1521 kb)

Supplementary material 8 (TIF 1521 kb)

## Abbreviations

ACLF: Acute-on-chronic liver failure
AUROC curve: Areas under the receiver operating characteristic curve
BCAA: Branched-chain amino acid
CP-A: Child–Pugh class A
CP-B: Child–Pugh class B
CP-C: Child–Pugh class C
DPPH: 1,1-Diphenyl-2-picrylhydrazyl
HBV: Hepatitis B virus
HCV: Hepatitis C virus
HMA: Human mercaptoalbumin
HNA: Human nonmercaptoalbumin
HOMA-IR: Homeostasis model assessment insulin resistance
HPLC: High-performance liquid chromatography
HSA: Human serum albumin
ROC: Receiver operating characteristic
