# Supplementary figures and images for: Oral branched-chain amino acid granules improve structure and function of human serum albumin in cirrhotic patients
Source: J Gastroenterol. 2016 Nov 21;52(6):754–65. doi: 10.1007/s00535-016-1281-2 (PMC5437197; doi:10.1007/s00535-016-1281-2)

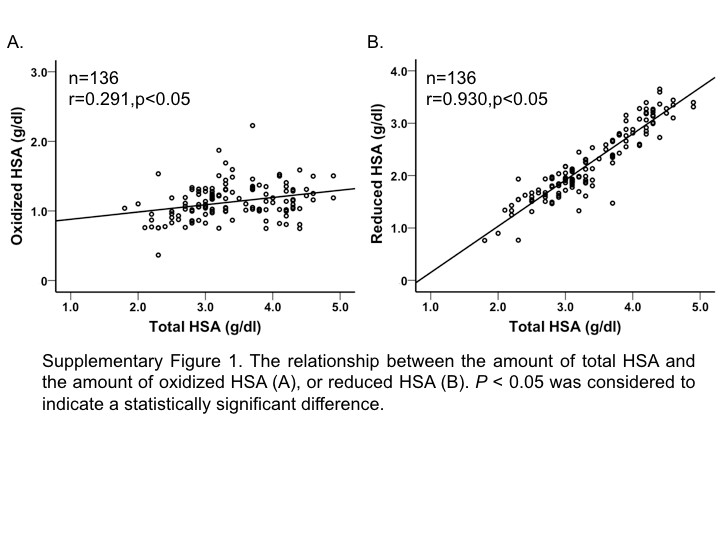

Supplement: Supplementary file 1 — Supplementary material 1 (TIF 86 kb) [file 535_2016_1281_MOESM1_ESM.tif]

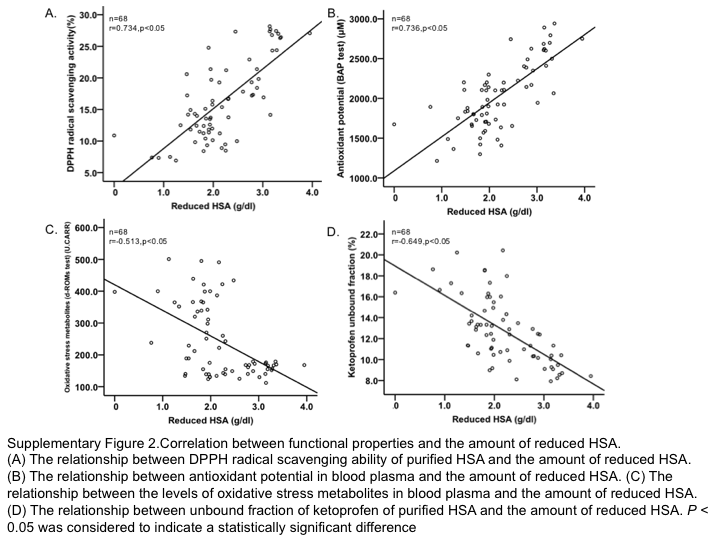

Supplement: Supplementary file 2 — Supplementary material 2 (TIF 137 kb) [file 535_2016_1281_MOESM2_ESM.tif]

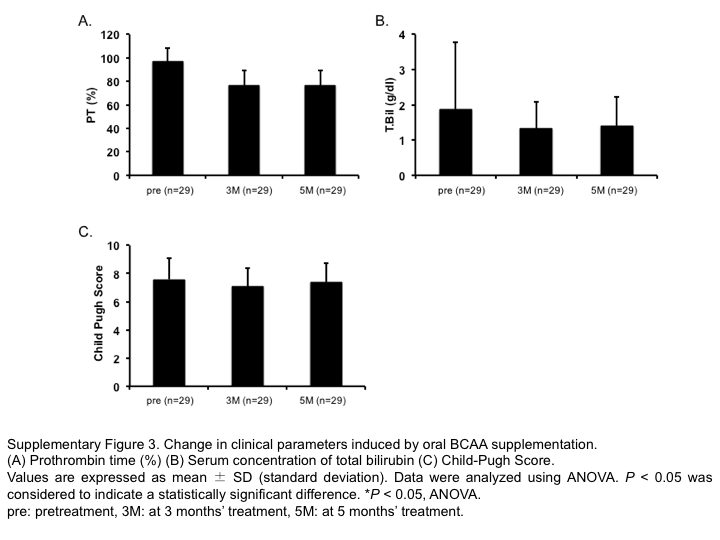

Supplement: Supplementary file 3 — Supplementary material 3 (TIF 85 kb) [file 535_2016_1281_MOESM3_ESM.tif]

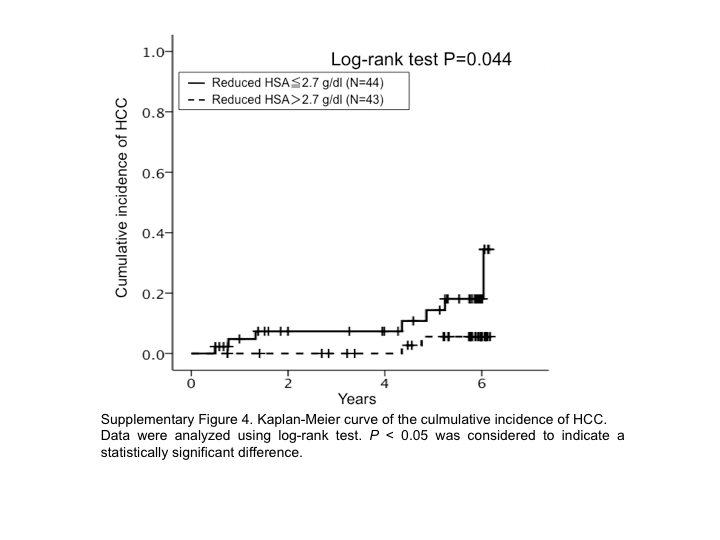

Supplement: Supplementary file 4 — Supplementary material 4 (TIF 58 kb) [file 535_2016_1281_MOESM4_ESM.tif]

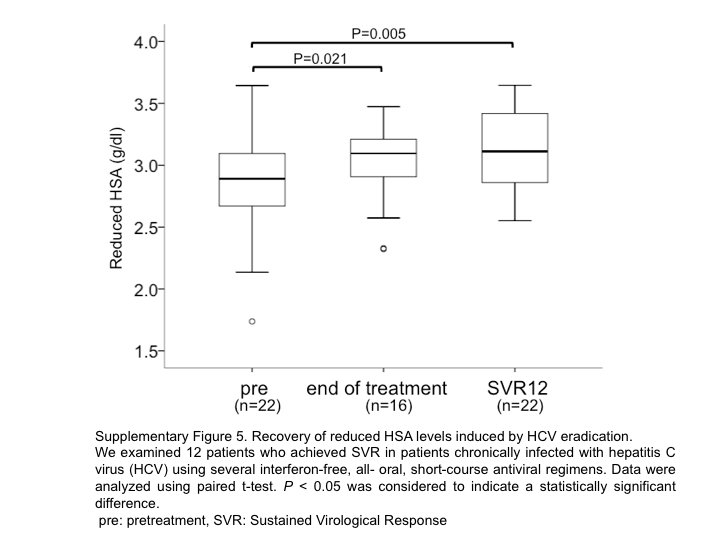

Supplement: Supplementary file 5 — Supplementary material 5 (TIF 77 kb) [file 535_2016_1281_MOESM5_ESM.tif]

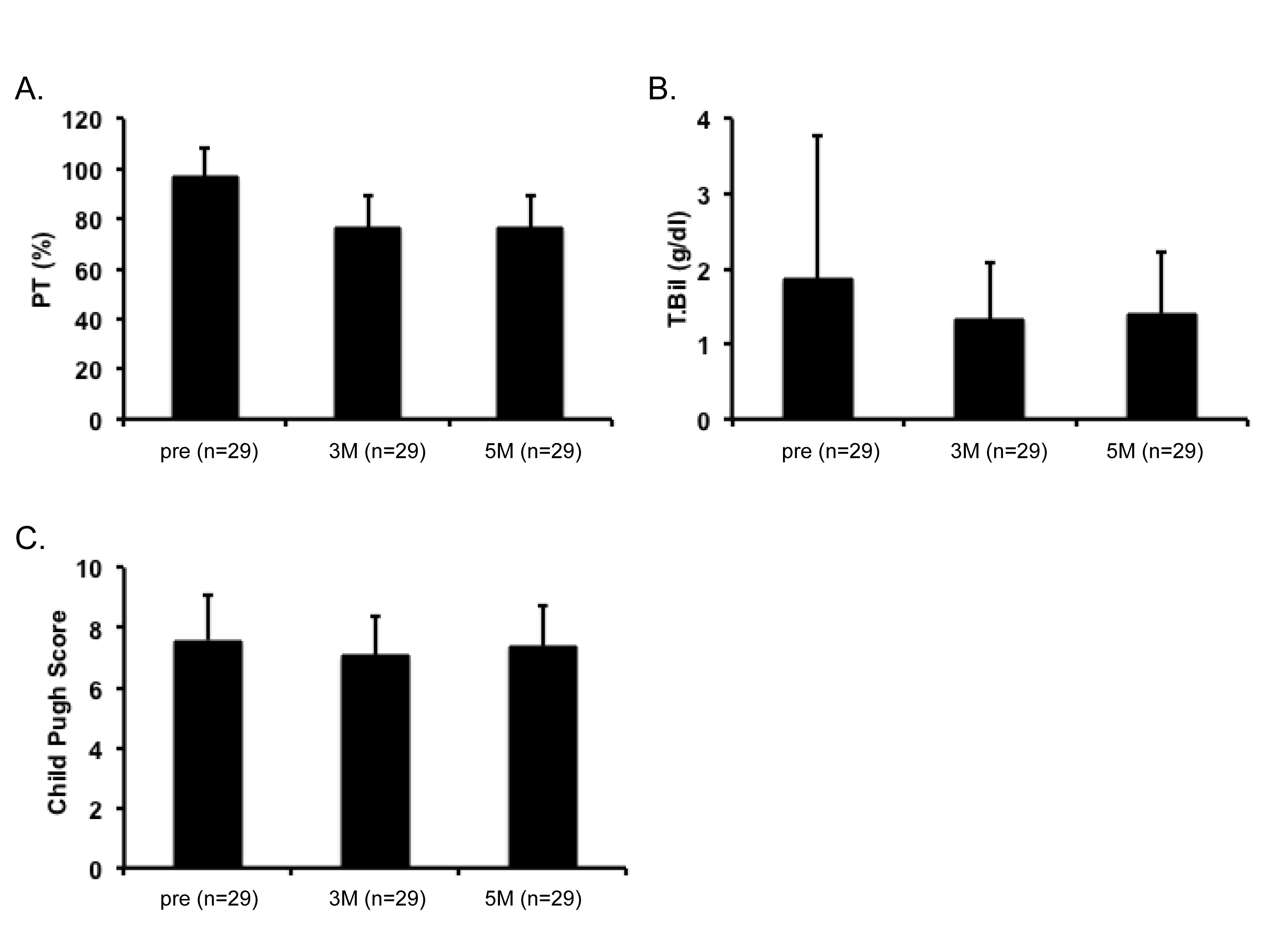

Supplement: Supplementary file 6 — Supplementary material 6 (TIF 7077 kb) [file 535_2016_1281_MOESM6_ESM.tif]

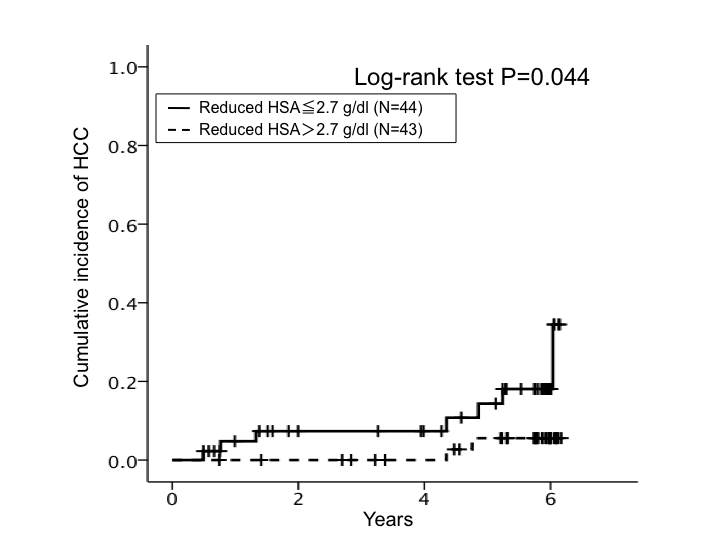

Supplement: Supplementary file 7 — Supplementary material 7 (TIF 1521 kb) [file 535_2016_1281_MOESM7_ESM.tif]

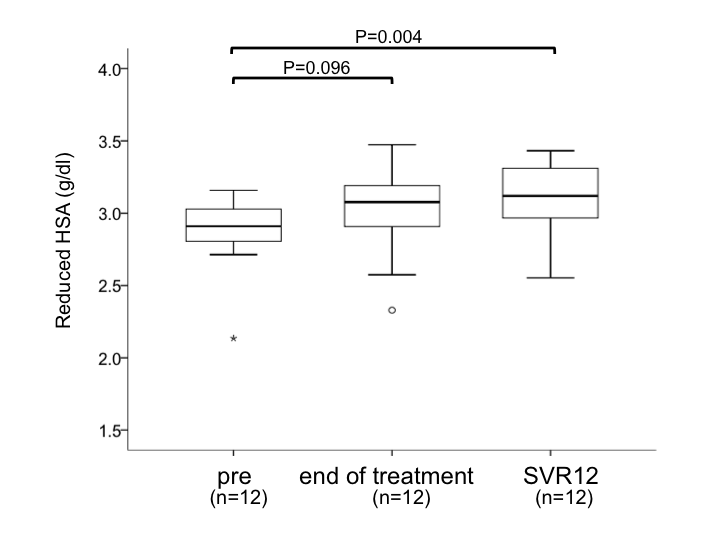

Supplement: Supplementary file 8 — Supplementary material 8 (TIF 1521 kb) [file 535_2016_1281_MOESM8_ESM.tif]
